# Antipyrin in Migraine

**Published:** 1888-04

**Authors:** 


### *ANTIPYRIN IN MIGRAINE.*

During the last two months, I have treated twenty case of migraine; several of the patients having suffered for over ten years, and, finding all drugs useless, had become reconciled to being periodically prostrated for one or two days. In every case, I ordered eight grains of antipyrin, dissolved in water or lemonade, to be repeated each half hour until cured, the patient to remain lying down. Most of the cases were quite cured by two powders, but the most obstinate yielded to three, and in no case did the antipyrin fail. A cup of warm tea sometimes seemed to help, and the only inconvenience due to the treatment was, in a few of the cases, considerable sweating.

Many of the patients can hardly credit that, instead of being utterly helpless for twenty-four hours, they can now cut short an attack in one hour.

There is another great advantage in using antipyrin, and that is, that it prevents as well as cures these attacks. One lady, who cannot remember having fewer attacks than three a month, each lasting about thirty-six hours, has been quite free for eight weeks, and this she attributes solely to the occasional use of an antipyrin powder.—*Weekly Med. Review.*

